# Address to the Graduating Class, Ohio College of Dental Surgery—Session 1860-’61

**Published:** 1861-03

**Authors:** J. Richardson


THE
### DENTAL REGISTER OF THE WEST.

VOL. XV.] MARCH, 1861. [No. 3.

### Original Essays and Communications.

### ADDRESS TO THE GRADUATING CLASS, OHIO COLLEGE OF DENTAL SURGERY—SESSION, 1860-61.

BY PROF. J. RICHARDSON.

GENTLEMEN GRADUATES:—By the official act of the venerable President of the Board of Trustees of this Institution, you have, this evening, been severally and formally invested with all the rights, dignities, and immunities which attach to the degree of Doctor in Dental Surgery. That this distinction has been fairly won and worthily bestowed, the signatures attached to the diplomas now in your possession sufficiently attest.

As the last official duty of those who have been more immediately charged with the responsibilities of your collegiate training, it becomes my privilege, speaking for myself and in behalf of my associates, to offer you our hearty congratulations upon an event of so much present and future interest to you, and to extend to each one a most friendly and cordial welcome into the ranks of the profession.

To us, who, during the time that is passed, have been the immediate and gratified witnesses of your uniformly courteous and gentlemanly bearing, and who have marked, from day to day, your earnest and unfaltering purposes in the line of your various duties, and who have been encouraged throughout by the evidences of advancement and proficiency which have rewarded their efforts to instruct, the privilege of participating in this formal and public recognition and endorsement of your claims to equal fellowship and privileges with the best and most honored in the profession, is especially gratifying.

With the exercises of this evening, the relations, heretofore existing between us as teachers and pupils, terminate. We remain to extend to others, who may hereafter seek instruction in this institution, the same assistance and encouragement that have been freely and cheerfully tendered to you. You, on the other hand, go hence to assume, in the locations which you may respectively choose, the varied duties and responsibilities of your new and untried vocation. As students under our charge, therefore, we no longer address you,—that immediate relation, voluntarily assumed by you in the first instance, and necessarily limited in its duration, no longer exists; you are now our *peers* in the profession. But the very act which creates you such and annuls your former relations, has also imposed new ones, not voluntary, nor conditional, nor of circumscribed duration, but absolute, binding, and perpetual—I mean those which attach to the condition of alumni. It is upon the duties growing out of your relations as graduates, that we propose to address you this evening before we part, some of us, it may be, forever.

Among the obligations of your future will be those which impose upon you the exercise of *good faith* toward your Alma Mater, and indeed it may be said that the strict fulfillment of all your duties and obligations to her, is but the summing up of all the requirements of your future professional lives. As her immediate representatives, we shall, in our own feeble way, advocate her claims to every consideration, in respect to your fidelity as alumni, which her own and the interests of the profession, whose minister she is, may rightfully exact of you.

In her own right, then, she may be esteemed not altogether unworthy of some consideration at your hands. Toward our natural parents, our hearts ever kindle with emotions of filial love, respect, and gratitude, and we carry with us, in the after struggle of life, the hallowed memories of the chilhood home made sacred by their love and solicitude. But among the cherished ones of earth, none so beloved and venerated as her whose ceaseless vigilance kept watch over the feebleness of our infancy and childhood ; she who directed our unfolding thoughts and impressed upon the tender and expanding mind and heart those precepts and principles which give character, strength, and nobility to manhood, and whose counsels point the way to useful distinction, and give assurance of an unblemished name as a heritage to the world when the weary strife is over. Worthy of all homage, love, respect and gratitude is she; and though every other image may be forgotten in the feverish, selfish struggle with the world, the memory of our mother never fades or passes away. Not so imperative or so exalted, perhaps, are the claims of your Alma Mater upon your remembrance, respect and gratitude, but, as your fostering mother, this institution may, of right, *demand* that she shall at least be *honored* in your future professional acts by a fearless, consistent, truthful discharge of all your relations as alumni. The Faculty, acting in behalf of the profession, and with an intelligent sense of their accountability, have assumed, in the full belief of your worthiness, the grave responsibility of endorsing your fitness and qualifications for the creditable discharge of the high and important functions of your calling, and have certified to your claims upon the recognition, respect, and fellowship of the fraternity. This, gentlemen, is no slight testimonial, and I hesitate not to say, that the recipient who fails to make the endorsement good before the profession and the world, by any subsequent acts tending to compromise his good name as a man or practitioner, betrays and dishonors his Alma Mater, and brings reproach upon her representatives.

It is not enough, in the discharge of your duties to the school, that you have cancelled such pecuniary obligations as have been stipulated for in the compact between us. We, in a great measure, have but enunciated the truths which have been worked out by the common brain of the profession, and while your immediate pecuniary indebtedness to us as mere preceptors have been fully cancelled, there stands a party behind us, whose claims upon you are immeasurably above those which may be expressed in dollars and cents, and which, as graduates, it should be the leading purpose of your future professional lives to repay with interest. Much, if not all, of what you have acquired touching your profession is yours only by the generosity of those who have preceded you. You have the knowledge of scarcely a single fact in dental practice that is yours in your own right of discovery; nor do you hold scarcely a single truth or principle within the range of dental science in the right of your own mental conception or elaboration.

We repeat, therefore, that every man who avails himself, for purposes selfish or humanitarian, of the accumulated experiences and teachings which the common labor of the profession has garnered up, incurs thereby a debt which, in all honor and honesty, he is under obligations to cancel by an unreserved consecration of his best powers to the good of the profession and the interests of humanity. His acquired knowledge is but a common fund which he holds in trust, and by all the exactions of good faith, he should make a good account of the trust reposed in him, and see that it is faithfully executed. Various means suggest themselves by which you may be enabled to make some fitting return for the great benefits you have received through the labors of those who, through all the changing fortunes of a comparatively new profession, have worked steadily and hopefully on to make it what it now is. Prominent among those means are self-culture and improvement. If you have, for a moment, pictured to yourselves lives of luxuriant ease in the practice of your chosen profession, disabuse your minds of what can only tend to enervate and demoralize you, and what must inevitably lead to bitter disappointments, if you have a single aspiration to excel. It involves hard labor if a man but aims to keep up with the profession, but I hold even this to be beneath the dignity of a man's reach in the practice of his calling. What if all were content with simply *keeping up* with the profession? All acting on that principle would bring our specialty at once to a dead halt. Each one content with what is already known, would end all progression. The true idea or philosophy of progress is illustrated in every man's endeavor to add, to the department of knowledge to which his calling relates, something not already known. Many there are who, with criminal slothfulness and indifference, play their ignoble part with no thought of the great demands of science or of humanity pressing on them; human machines, vitalized with mere selfish instincts, who never move in any enterprise outside of the contracted circle of their own sordid interests, unless, perchance, they are driven to it with whip and spur; men who drivel over their daily round of office engagements and forced duties with the persistence and exclusiveness of an ass on a treadmill, and who make about as much progress in their profession as the aforesaid animal on the wheel, whose revolutions never advance his nose one solitary inch beyond the rack at which he feeds. Spiritless and indolent, they shrink like cravens from the more exalted duties of their position, and are seemingly content to eke out an inglorious and almost profitless life as dull and unproductive plodders in the beaten path, feeding from day to day, like professional mendicants, upon the intellectual crumbs that fall by the way from other men's brains.

Now, although it may not be altogether unworthy of a man's ambition o attain to a full knowledge of the facts and truths of his profession, yet it is certainly more worthy of his powers to take a step in advance of its present attainments, and to strive to enlarge its boundaries and augment its achievements by his own personal contributions. To do this it will be necessary for you to avail yourselves of all the means of self-improvement within your reach. You should be hard and unremitting students. You should read much, not for pastime or amusement, but for instruction. Read carefully and analytically; note well the *facts* presented to your minds, and place authors' inferences or deductions from those facts on trial. Facts you may not dispute, but opinions may be fairly challenged. Take no man's conclusions for granted, but subject them, in candor and with impartiality, to the test of your own judgment, observation and experience. Cultivate a spirit of independent thinking, but with modesty and distrust in your own infallibility. Nothing so surely tends to mental decrepitude as a slavish reliance upon the opinions of others,—nothing so fatal to mental growth and culture as the poor-spirited committal of your own judgment into the keeping of others. Treat every man's opinion with respect for your brother's sake, and while you do your own thinking, be always as ready to renounce errors of judgment when apparent to your consciousness, as you are to adopt those facts which commend themselves to your acceptance.

Prominent among the means for self-improvement, are dental periodicals. They are, in an eminent degree, worthy of our support and encouragement, and with an annual expenditure of less than twenty dollars, one may command every American journal devoted to dental science and art now published. They are the exponents, quarterly or monthly, of dentistry *as it is*, and they come to us teeming with the fresh experiences of every-day practice like ever gushing well-springs of pure water to slake our thirst for knowledge. They are the great heart of our literature, which, through innumerable ramifications, send their vitalizing influences into every tissue of the professional organism, quickening all parts with new life and powers, and contributing more than any other instrumentality to the wonderful growth and progress of the profession. You can not afford to be without these aids,—no investment will afford you such ample returns. To ignore them is to shut out the light from your laboratory and operating room. Without them, you will be distanced by competitors with half your capacities and industry. In this connection, allow me to suggest that the mere subscription support which you may give to these journals does not wholly fulfill your obligations. When you have paid the subscription price, you have only settled with the publishers. Members of the profession who fill page after page with the rich stores of their daily observations and experience, that you and I may be profited thereby, have a right to expect the same generous offering of our gifts upon a common altar for the common good, and you will most assuredly be wanting in good faith to them, and recreant to your manifest obligations, if you fail to respond, in a like spirit of liberality, to their equitable demands. To omit so plain a duty would be very like living in charity upon your associates.

The stated gatherings of the profession in conventions, local, State, and national, afford, also, distinguished opportunities for improvement. To these convocations, like devout pilgrims to some holy shrine, the liberal-minded men of the profession everywhere wend their way with their precious bequests of ripe experiences and carefully selected facts, to lay their gifts upon a common altar. From the multitude of offerings presented, some are scattered as chaff before the winds, while the grains, which remain to nourish and strengthen, are carefully gathered up and garnered, and anon are spread broadcast, to take root in productive soils, springing forth again with an hundred-fold increase to bless mankind. These conventions and societies are, indeed, in a most eminent degree, the touch-stones that test, with the certainty, almost, of infallibility, the validity and truth of every important fact and principle which concern us in practice If errors are rendered plausible by specious speculations and dangerous sophistries, the keen dissections of verbal and open discussions expose their nakedness; if rendered attractive by captivating oratory, they are stripped of their claims and pretensions by the inexorable logic of every-day experience. Thus, error is consumed, and from its ashes new lights spring forth, radiant with beauty, and enduring as truth itself. In these conventions many a priceless thought, under the inspiration of the moment, has come bubbling up to the surface, all unconsciously perhaps, and of obscure parentage it may be, but born to be clothed hereafter with immortality. Here the grim visaged spirits of jealousy, envy, and distrust are exorcised, while old friendships are renewed and new ones formed, binding and cementing all together in one common, compact brotherhood. Here the arrogant and inflated pretensions and assumptions of addle-brained gasconaders have their wings clipped and their masks removed; while the timid are re-assured, and modest merit is lifted up and patronized. Our conventions are emphatically schools for improvement, where every man is at once instructor and scholar, and no one may withhold himself from them without serious self-detriment and loss of personal weight and character in his profession.

Prominent among the educational facilities of the day, let us not pass unnoticed Dental Colleges. Their claims upon the consideration of the profession are too well-grounded and too generally recognized, to require any extended vindication at our hands. To you, as a portion of her offspring whom it has been her privilege and her pleasure to honor, this Institution trustingly commits her reputation and her interests. No better guaranty of your appreciation of the benefits to be derived from a regular course of collegiate instruction is required than the completion of your term of pupilage under her guidance and direction. We only ask, speaking for the cause of dental education everywhere, that you may encourage the enterprise by substantial tokens of your approval and appreciation, and that you may contribute to her support and prosperity by every means which you may rightfully exercise in her behalf. There is plain and palpable dereliction of duty, in this matter of college patronage, fairly chargeable to the profession of the West. The great Mississippi Valley is, to-day, teeming with probably over two thousand dentists, every man of whom has a personal and direct interest in all that pertains to, or affects, the character and reputation of the profession, and yet this school goes begging from year to year for countenance and patronage. No unfaithfulness to the trust reposed in them, or incompetency in the discharge of their allotted duties, has ever been charged upon the Faculties who have struggled on almost against hope, and yet this amphitheater, session after session, displays but a "beggarly account of empty seats." It would be affectation in me not to declare that the institution is worthy of better fortunes, and that she is more deserving at the hands of those whose interests, in every aspect of professional life, are identical with hers. There are scores upon scores who are loud-mouthed in denunciations of quacks and quackery, but are as tongue-tied as mutes in defense of the very means which, more than all others, give tone and respectability to their calling, and which, more than any other agency, is potent to crush out pretenders and mountebanks. We trust and believe, gentlemen, that you at least, with other alumni who have gone before, will yield a ready and hearty response to the claims of the Institution that has received and nourished you, and whose prosperity and usefulness in the future can not be indifferent to you.

I have, thus far, indicated to you, imperfectly perhaps, some of the leading obligations of your professional lives. There are many other aspects of the subject, bearing upon the duties and relations which you must assume, but I must hasten to a close.

In a few brief minutes we must part, and it will be in the cheering and confident assurance that your purposes lie in the right direction, and that your hands, your hearts, and your minds are prepared and nerved for the exigencies of the untried future. Between the cheering visions of that future, so natural to the hopefulness of our natures, and their realization, there is a long period fraught with many a joy and sorrow, which, for wise purposes, are hidden from our view by an impenetrable veil. I know, as do all who have passed through their term of pupilage, something of the student's dreams of after-life,—of the bright, enchanting, hopeful visions that crowd upon his awakened imagination as he looks with impatient gaze beyond the dull and plodding present into the glorious future. It is but the natural inspiration of the artless and aspiring soul before it is made sour and distrustful by the experiences of actual life. And it is well, for of that inspiration are born promptings and impulses, motives of thought and action, which impel him to take hold more cheerfully, more hopefully, and more courageously upon the work of the present. On that inspiration are laid those foundations, broad and deep, that shall prevail when trials, and disappointments, and temptations come, if come they must.

Thus far, it has been plain sailing with you. No false promptings of self-interest have yet lured you into those waters beneath whose placid bosom treacherous rocks lie concealed, and on which many a goodly craft, well-manned, has struck, and parted, and gone down. No bitter disappointments and heart-sickenings that wait upon "hope deferred," have yet hung like leaden weights upon life's first enterprise. No bitter experiences of neglect, indifference, or ingratitude, that blast like mildew, have yet been yours to experience. All has been calm and peaceful drifting, with the music of rippling waves beneath, and the brightness of a cloudless sky above. And though I would not willingly shut out from your gaze the vision of that bright blue sky by clothing it with frowning and portentous clouds, nor drown the sweet music of those gently murmuring waves by the shrill cry of "breakers ahead," yet if word of mine may strengthen you in the hour of need, I would not see you exposed to the danger of drifting, unconsciously it may be, into those treacherous waters in whose dark depths many an unwary voyager has been engulfed.

I say it not to discourage, gentlemen, but rather to fortify you against what is inevitable,—your coming and immediate future will be full of perils to your personal and professional integrity. The first few years of your active professional lives will, in all human probability, either make or mar you for all coming time. Like base metals, you will be consumed in the fire, or as gold refined in the furnace, you will come out from the ordeal not only unscathed, but strengthened and ennobled by the trial of your integrity. Many, very many vindicate their manhood at all hazards and against all odds, and stand proudly unimpeached before the world;—others lapse and are lost. The history of every school devoted to the teaching of Medical or Dental Science is, doubtless, not wanting in examples of shameless recreancy to the obligations which their relations as alumni impose. Such instances I am happy to believe are rare among the graduates of this school, though I have clearly in my mind's eye to-night, one or more who, with the honors of the school fresh upon them, have descended to such practices as are scarcely paralleled by the veriest mountebank that hawks his secret nostrums in the public squares. But it is a cheering and hopeful evidence of the profession's appreciation of the charlatan's tricks and arts, that the parties who have thus prostituted their acquirements and betrayed the generous confidence reposed in them, do not, to-day, enjoy either the profession's respect, sympathy, or recognition. Their histories are those of powers perverted and manhood sacrificed to compass ends which center wholly in self-interest, and as such they are introduced to "point a moral."

And now, gentlemen, with sentiments of the highest regard for you personally, and an earnest hope and desire that the success of your future professional lives may be such as shall add luster to your names, and confer nameless blessings upon mankind, whose sufferings and misfortunes it will be your peculiar province to ameliorate, we bid you a reluctant farewell.

